# Cloudgene: A graphical execution platform for MapReduce programs on private and public clouds

**DOI:** 10.1186/1471-2105-13-200

**Published:** 2012-08-13

**Authors:** Sebastian Schönherr, Lukas Forer, Hansi Weißensteiner, Florian Kronenberg, Günther Specht, Anita Kloss-Brandstätter

**Affiliations:** 1 Division of Genetic Epidemiology; Department of Medical Genetics, Molecular and Clinical Pharmacology, Innsbruck Medical University, Innsbruck, Austria; 2 Department of Database and Information Systems; Institute of Computer Science, University of Innsbruck, Innsbruck, Austria

## Abstract

**Background:**

The MapReduce framework enables a scalable processing and analyzing of large datasets by distributing the computational load on connected computer nodes, referred to as a cluster. In Bioinformatics, MapReduce has already been adopted to various case scenarios such as mapping next generation sequencing data to a reference genome, finding SNPs from short read data or matching strings in genotype files. Nevertheless, tasks like installing and maintaining MapReduce on a cluster system, importing data into its distributed file system or executing MapReduce programs require advanced knowledge in computer science and could thus prevent scientists from usage of currently available and useful software solutions.

**Results:**

Here we present Cloudgene, a freely available platform to improve the usability of MapReduce programs in Bioinformatics by providing a graphical user interface for the execution, the import and export of data and the reproducibility of workflows on in-house (private clouds) and rented clusters (public clouds). The aim of Cloudgene is to build a standardized graphical execution environment for currently available and future MapReduce programs, which can all be integrated by using its plug-in interface. Since Cloudgene can be executed on private clusters, sensitive datasets can be kept in house at all time and data transfer times are therefore minimized.

**Conclusions:**

Our results show that MapReduce programs can be integrated into Cloudgene with little effort and without adding any computational overhead to existing programs. This platform gives developers the opportunity to focus on the actual implementation task and provides scientists a platform with the aim to hide the complexity of MapReduce. In addition to MapReduce programs, Cloudgene can also be used to launch predefined systems (e.g. Cloud BioLinux, RStudio) in public clouds. Currently, five different bioinformatic programs using MapReduce and two systems are integrated and have been successfully deployed. Cloudgene is freely available at http://cloudgene.uibk.ac.at.

## Background

Computer science is becoming increasingly important in today’s genetic research. The accelerated progress in molecular biological technologies puts increasing demands on adequate software solutions. This is especially true for next generation sequencing (NGS) where costs are falling faster than for computer hardware [1]. As a consequence, the accompanying growth of data results in longer execution times of currently available programs and requires new strategies to process data efficiently. The MapReduce framework [2] and especially its open-source implementation Hadoop [3] has become more and more popular for processing and analyzing terabytes of data: Mapping NGS data to the human genome [4], calculating differential gene expression in RNA-seq datasets [5] or even simpler but time intensive tasks like matching strings in large genotype files^1^ are already successfully implemented scenarios. With MapReduce, a computation is distributed and executed in parallel over all computer nodes in a cluster, allowing to add or to remove nodes on demand (scale-out principle). The developer is responsible to write the corresponding map and reduce task and the framework itself is taking over the parallelization, fault tolerance of hardware and software, replication and I/O scheduling. Unfortunately, small to medium sized genetic research institutes can often hardly afford the acquirement and maintenance of own computer clusters. An alternative is public cloud computing which offers the possibility to rent computer hardware from different providers like Amazon’s Elastic Compute Cloud ( http://aws.amazon.com/ec2/) on demand.

Working with cluster architectures requires a background in computer science for both setting up a cluster infrastructure and executing MapReduce programs, where sometimes a graphical user interface (GUI) is lacking at all. To improve usability, different programs [4-6] have been developed with a focus on a simplified execution. This constitutes a major improvement for scientists with the down side to implement a new GUI for every future MapReduce program. Additionally, concatenating different programs to a pipeline is still hampered.

In this paper we present Cloudgene, a platform to integrate available MapReduce programs via manifest files and to facilitate the use of on-demand cluster architectures in cloud environments. Cloudgene’s biggest advantage lies in simplifying the import and export of data, execution and monitoring of MapReduce programs on in-house (private clouds) or rented clusters (public clouds) and allowing the reproducibility of an analysis or analysis pipeline.

## Implementation

### Overall design

**Figure 1:**
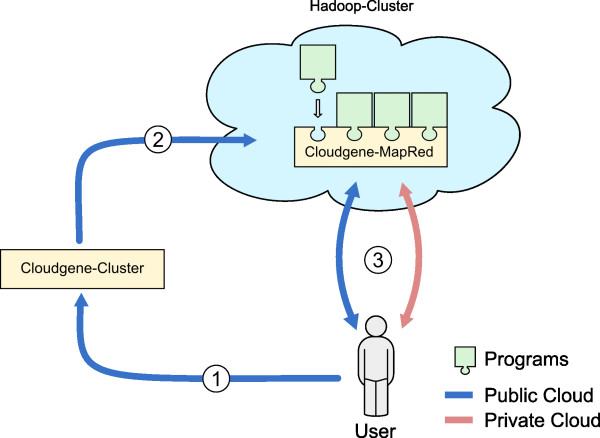
**The use of Cloudgene in public or private clouds.** When using Cloudgene in a public cloud (blue path), the first module Cloudgene-Cluster launches a cluster in the cloud (step 1) and installs all necessary data for the specific scenario including the second module Cloudgene-MapRed (step 2). When finished, the user is able to communicate with the cluster and to execute and monitor jobs from plugged in MapReduce programs (step 3). In addition, Cloudgene-MapRed can also be used stand-alone on an in-house cluster (private cloud), with the precondition that a running Hadoop Cluster is available (red path, only step 3 is necessary).

In order to be used in public and private clouds, Cloudgene consists of two independent modules, Cloudgene-Cluster and Cloudgene-MapRed. Cloudgene-Cluster enables scientists to instantiate a cluster on a public cloud, currently applied on Amazon’s EC2. The end user is guided through the configuration process via graphical wizards, specifying all necessary cluster information including the complete hardware specification, security credentials and SSH keys. Cloudgene-MapRed can be seen as an additional layer between Apache Hadoop and the end user and defines a user-friendly way to execute and to monitor MapReduce programs, providing a standardized import/export interface for large datasets. Cloudgene-MapRed supports the execution of Hadoop jar files (written in Java), the Hadoop Streaming mode (written in any other programming language) and allows a concatenation of programs to program pipelines. One central idea behind Cloudgene is to integrate available and future programs with little effort: Therefore, Cloudgene specifies a manifest file (i.e. configuration file) for every program which defines the graphical wizards to launch a public cluster or MapReduce jobs (see section ‘Plug-in interface’). Figure 1 summarizes how these two modules collaborate together to execute programs depending on the specified cluster environment.

### Architecture and technologies

**Figure 2:**
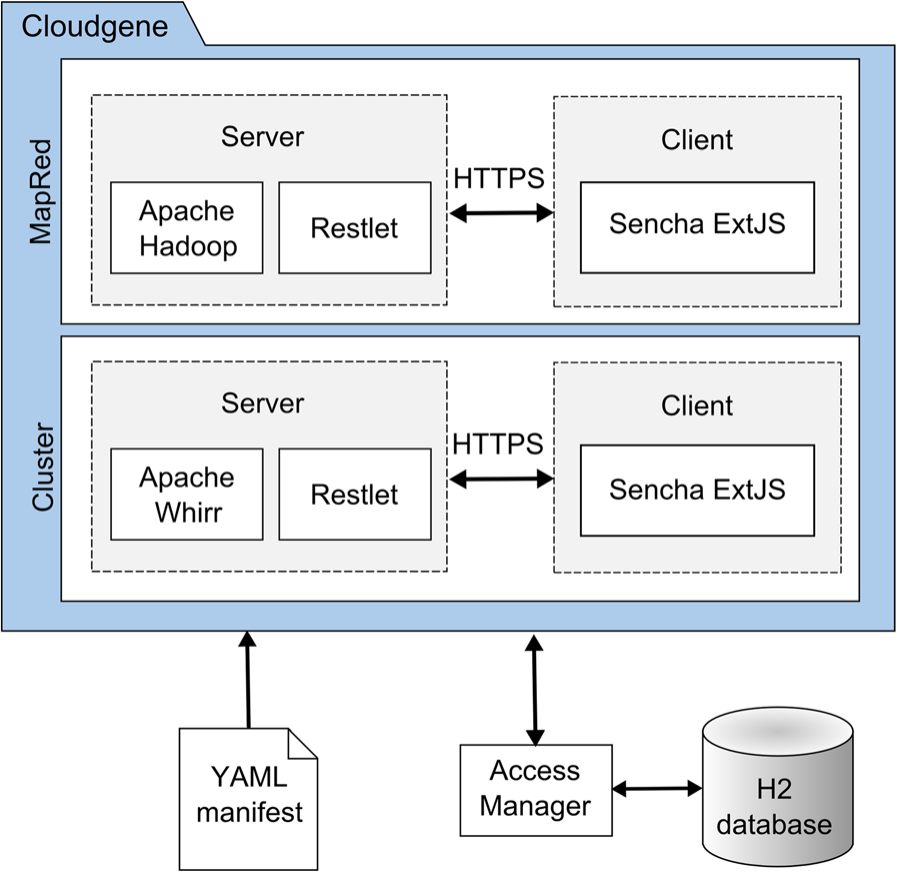
**Cloudgene system architecture.** Cloudgene consists of two independent modules, Cloudgene-Cluster and Cloudgene-MapRed. Both implement a client–server architecture using open source technologies. The client is implemented in JavaScript utilizing Sencha Ext JS and communicates with the Restlet server via a secured connection (HTTPS). The program parameters are read out from its manifest file written in YAML. Both modules are username/password secured and store all required metadata in the relational SQL database H2.

Both modules are based on a client–server architecture: The client is designed as a web application utilizing the JavaScript framework Sencha Ext JS ( http://www.sencha.com). On server side all necessary resources are implemented in Java by using the RESTful web framework Restlet ( http://www.restlet.org/) [7]. The communication between client and server is obtained through asynchronous HTTP requests (AJAX) with JSON ( http://json.org) as an interchange format. Cloudgene is multi-user capable and encrypts the transmission between server and client with HTTPS (Hypertext Transfer Protocol Secure). To integrate new programs and describe all properties of a program or program pipeline, the YAML ( http://www.yaml.org) format is used to define the manifest file. All required metadata is stored in an embedded Java SQL database H2 ( http://www.h2database.com). The Apache Whirr [8] project is used to launch a cluster on Amazon EC2, to combine nodes to a working MapReduce cluster and to define the hardware environment of it. Figure 2 summarizes the overall architecture.

### Cloudgene-Cluster

**Figure 3:**
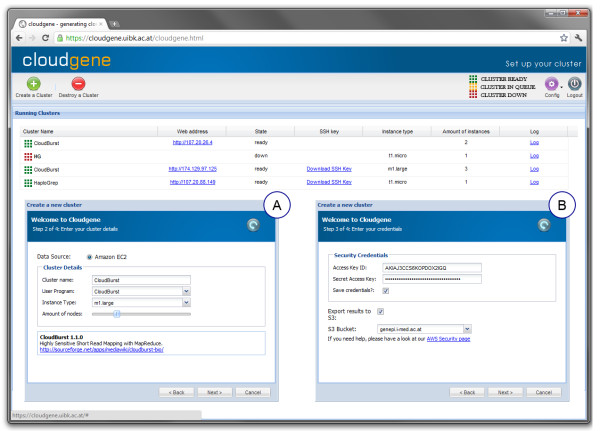
**Screenshot of Cloudgene-Cluster.** Cloudgene-Cluster allows launching a cluster and setting up the cloud environment. The main window displays all cluster configurations and indicates their current status. Subfigures (**A**) and (**B**) show the necessary wizard steps where the program itself and its parameters can be selected. All needed software is installed automatically and the end user receives an URL of the cluster namenode where Cloudgene-MapRed has been installed.

After a successful login to Cloudgene-Cluster, the main window provides the possibility to create or to shut down a public cluster and to get an overview of all previously started nodes (Figure 3). When launching a new cluster a wizard is shown: in a first step the cloud provider, cluster name, the program to install, the amount of nodes and an available instance type (i.e. hardware specification of a node) need to be selected (Figure 3A). Subsequently, the cloud security credentials have to be entered and an SSH key has to be chosen or uploaded (Figure 3B). For user convenience, security credentials need only be entered once for every session (until log-out) and can additionally be stored encrypted in the H2 database. A storage of SSH keys is especially useful for advanced users who want to login into a node via an SSH console. In addition, an S3 bucket can be predefined for an automatic transfer of MapReduce results. Within minutes a ready-to-use cluster is created, where all necessary software is installed and parameters are set. As a final step, Cloudgene-Cluster installs Cloudgene-MapRed on the launched cluster and returns the web address for accessing it. Cloudgene-Cluster provides the possibility to download SSH keys, to access the log with all performed actions during cluster setup, to add new users or to logout from the system.

### Cloudgene-MapRed

**Figure 4:**
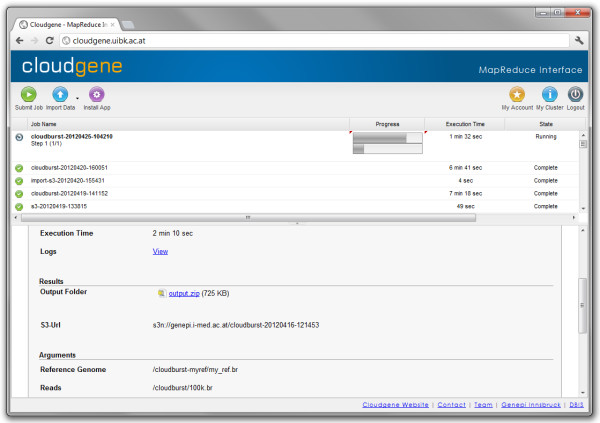
**Screenshot of Cloudgene-MapRed.** Cloudgene-MapRed runs on the namenode of a Hadoop cluster and enables a simplified execution of MapReduce jobs via a web interface. All input/output parameters can be easily set through wizards. Cloudgene-MapRed shows the currently executed jobs, visualizes the progress of MapReduce jobs, receives feedback and allows viewing and downloading results.

The main window of Cloudgene-MapRed (Figure 4) is structured as follows: The toolbar on top contains buttons for program (job) submission, data import and program installation. Additionally, buttons for changing the account details (security credentials, general information and S3 export location for results) and detailed cluster information (e.g. number of nodes, MapReduce configuration) are provided. All currently running and finalized jobs including name, progress, execution time and state are displayed in the upper panel. For running jobs, the progress of the map and reduce phases are displayed separately. The lower panel displays the job-specific information including input/output parameters, S3 export location, job arguments, execution time and results. The export location is created automatically using the naming convention *S3bucket/jobname/timestamp*. Moreover, the detail view contains a link to the logfile in case of errors.

Before launching a new job, data needs to be imported into the distributed file system (Hadoop Distributed File System), whereby the data source has to be selected. Currently, Cloudgene supports a data import from FTP, HTTP, Amazon S3 buckets or direct file uploads. A job can be submitted by specifying the previously imported data and the program-specific parameters. After launching a program, the process can be monitored and all jobs including results are viewable or downloadable. As all data on a cluster in a public cloud are lost on shutdown, Cloudgene automatically exports all results, log data and if specified also imported datasets in parallel to an S3 bucket.

### Plug-in interface

**Figure 5:**
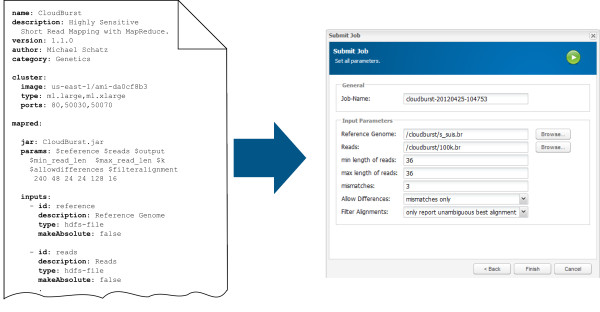
**A standard YAML manifest file.** The manifest file contains a metadata block (name, description, category and website) and a logical block for both Cloudgene modules, including all parameters that are necessary to execute the program and from a user’s view often hard to decide. This file has to be written once and is provided to scientists. Cloudgene checks at every start up for new programs and designs the web interface dynamically to the specific scenario. Here, the submit form for CloudBurst has been generated with information from the manifest file.

To integrate new programs into Cloudgene, a simple structured YAML manifest file has to be specified including a section for both Cloudgene-Cluster and Cloudgene-MapRed. This manifest file needs to be written once and can be either provided to other scientists by the developer or written by any person who is familiar with the execution of a MapReduce program. The manifest file starts with a block containing general program information (e.g. name, author, description, webpage). In the Cloudgene-Cluster section the file system image, available instance types, firewall settings, services (e.g. MapReduce), installation scripts (additional software to install) and other program depended parameters are specified. The Cloudgene-MapRed section contains all necessary information that characterizes a MapReduce program including input and output parameters or Cloudgene’s step functionality (i.e. job pipelining). At start up, Cloudgene loads all necessary information from the manifest file and generates the program specific wizards. Figure 5 shows the integration of CloudBurst into Cloudgene-MapRed. To simplify the integration process for end users, all currently tested MapReduce programs including working manifest files, a detailed description of available parameters, available instance types and a tutorial on how to set up an EC2 security credentials can be found on our website. Furthermore, Cloudgene-MapRed provides with its integrated web repository a mechanism to install currently available programs directly via the web interface.

## Results

**Figure 6:**
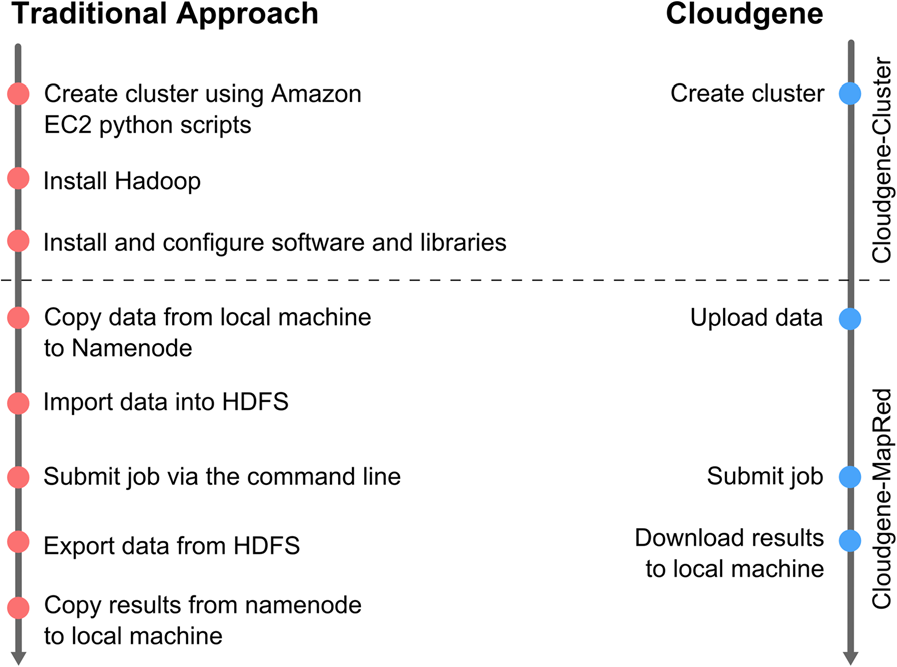
**Comparison of approaches.** All necessary steps to create a cluster and run a job can be executed and monitored via Cloudgene yielding to a significant simplification compared to a traditional approach. No command line is needed and complicated tasks are hidden from the end user at any time.

Cloudgene’s overall aim is to simplify the process of executing MapReduce programs including all required steps on private and public clouds (Figure 6). In the following section, we want to show on different case scenarios the diversity and advantage of Cloudgene. Table 1 summarizes all currently integrated programs.

**Table 1:** Currently integrated programs

	**Name**	**Details**
**MapReduce programs**	CloudBurst [4]	Highly sensitive short read mapping with MapReduce.
	Myrna [5]	A cloud computing tool for calculating differential gene expression in large RNA-seq datasets.
	Crossbow [6]	A scalable software pipeline for whole genome re-sequencing analysis.
	FastQ-Preprocessing^4^	Quality control for high throughput sequence data in fastq format.
	SNPFinder^4^	Filters and extracts certain SNPs from genome wide association studies datasets.
	Hadoop-Examples [3]	Several Hadoop example applications including Sort and Grep.
**System images**	Cloud BioLinux [10]	An AWS-EC2 image that includes a wide range of biological software, programming libraries as well as data sets.
	RStudio [11]	An AWS-EC2 image that enables the usage of all R programming tools via a web interface.
**Web applications**	HaploGrep [13]	A web application to determine mitochondrial DNA haplogroups.

### Integrating existing MapReduce programs

As mentioned above, several programs (CloudBurst [4], Myrna [5] or Crossbow [6]) already exist implementing a MapReduce approach to process data. To demonstrate the benefit of Cloudgene, we integrated these programs by writing appropriate manifest files, including sections for Cloudgene-Cluster (public clouds) and Cloudgene-MapRed (private and public clouds). In case of CloudBurst, a MapReduce job can now be executed graphically and the benchmark tests show that Cloudgene is scalable in time and competitive to Amazon’s Elastic MapReduce platform [9] regarding to cluster setup and program execution time (see Table 2 for a detailed comparison). For Myrna and Crossbow, a web interface has already been made available by the authors using Hadoop’s streaming mode. Nevertheless, by integrating these programs into Cloudgene, the users still benefit from (1) a standardized way to import/export data, (2) a system which keeps track of all previous executed workflows including the complete configuration setup (input/output parameters, execution times, results) and (3) the possibility to concatenate different MapReduce jobs to pipelines. Here, Cloudgene’s pipeline functionality (specified as ‘steps’ in the manifest file) has been used to execute several computation steps of Crossbow and Myrna. This can be achieved by defining the output directory of step *x* (e.g. step 1: Pre-processing) as the new input directory for step *x + 1* (e.g. step 2: Alignment) in the manifest file. Even if the newly created workflow consists of several steps in the manifest file, the user can start it as one job. Additional file 1 includes the corresponding manifest files showing the step functionality in detail.

**Table 2:** **Wall-Times: Cloudgene compared with Amazon Elastic Map-Reduce executing CloudBurst with input data from *s3n://elasticmapreduce/samples/cloudburst***

*Wall Times*			
**EC2-Nodes**	**Instance type**	**Cloudgene**	**Amazon EMR**
2 + 1	m1.small	21 min	25 min
4 + 1	m1.small	17 min	18 min
8 + 1	m1.small	12 min	12 min
*Import Data Times*			
**Type**	**Source**	**Data Volume**	**Time**
FTP	http://ftp.1000genomes.ebi.ac.uk/vol1/ftp/data/HG00096/sequence_read	~18 GByte	28 min
S3-Bucket	s3n://1000genomes/data/HG00096/sequence_read	~18 GByte	11 min

### Integrating novel MapReduce programs

For a proof of concept, we implemented a MapReduce pipeline for FastQ pre-processing to check the quality of large NGS datasets similar to the FastQC tool^2^, in which statistics like the sequence quality, base quality, length distribution, and sequence duplication levels are calculated. Unlike FastQC, which can be executed on a single computer and where due to memory requirements only a sequence subset is used for quality control computations, our implementation has the advantage that the complete set of sequences is included into the statistical analysis. After processing all sequences, a consecutive program transforms the results into meaningful plots and can finally be downloaded as pdf-files. Again, this shows the step functionality of Cloudgene-MapRed in which different programs can be connected to a pipeline (i.e. step 1: calculating statistics, step 2: generate plot). Moreover, fastq input-files can be imported from public Amazon S3 buckets (e.g. raw data from the 1000 genomes public S3 bucket), which is especially useful in combination with Amazon EC2 since data transfer from S3 to EC2 nodes is optimized (see Table 2 for measurements of import times).

A further scenario for a simple but time-intensive task is the filtering and extracting of certain rows from a large SNP-genotype file as usually used in genome-wide association studies with a file size of several gigabytes. Again, this case scenario was implemented as a MapReduce job by the authors of this paper and has been integrated into Cloudgene.

All integrated and introduced programs are available for download on our webpage or can be installed from the Cloudgene repository directly.

### Launching a stand-alone image

Different file system images (called AMIs in Amazon terminology) exist to provide a convenient way for setting up systems in the cloud: Cloud BioLinux [10] is a suitable file system image in Bioinformatics including a wide range of biological software, programming libraries as well as data and is therefore an excellent basis for bioinformatic computations in the cloud. RStudio [11] is a development environment for R, available as an EC2 image [12] and allows the usage of all R programming tools via a web interface. Both systems have been successfully integrated into Cloudgene enabling scientists to launch these systems via Cloudgene-Cluster. Cloud BioLinux has further been used as the underlying image for the integration of Myrna and Crossbow into Cloudgene, since most of the required software is already installed and the cluster installation process is therefore simplified.

### Launching a web application

Besides the mentioned MapReduce scenarios, Cloudgene can also be used to host a user defined web application on a public cloud. We demonstrated this on HaploGrep [13], a tool to determine mitochondrial DNA haplogroups from mtDNA profiles. Especially for large input data, HaploGrep requires a lot of main memory, thereby making a public cloud node with sufficient main memory an adequate choice. For this purpose a simple shell script was integrated into the setup process to start the HaploGrep web server. This shell script can be defined in the manifest file of Cloudgene-Cluster and is executed automatically on each cluster node.

## Discussion

Although MapReduce enabled a scalable way to process and analyze data, the execution of programs and the overall setup of cluster architectures still includes non-trivial tasks and hampers the spread of MapReduce programs in Bioinformatics. We therefore developed and implemented Cloudgene, which provides scientists a graphical execution platform and a standardized way to manage large-scale bioinformatic projects.

### Strengths and limitations

The usage of Cloudgene has several strengths: (1) Programs can be executed via one centralized platform, thereby standardizing the import/export of data, the execution and monitoring of MapReduce jobs and the reproducibility of programs or new defined program pipelines; (2) scientists can decide flexibly in which environment (public or private cluster) a program should be executed depending on the case scenario to guarantee an appropriate level of data security and to reduce data transfer times; (3) an Amazon EC2 cluster can be launched via the Cloudgene web interface, thus simplifying the overall setup and making Cloudgene available in a public cloud; (4) new MapReduce programs can be integrated by using Cloudgene’s plug-in interface without changing the program code at all.

However, Cloudgene has limitations as well: (1) since the main focus of our approach lies on a simplified execution of MapReduce jobs, programs using other paradigms (e.g. MPI or iterative processing) are currently not supported; (2) Cloudgene allows the concatenation of jobs to simple pipelines with the limitation that pipelines have to be executed from start to end and currently does not allow the execution of specific pipeline steps automatically (e.g. restart at step 3); (3) since the amount of included cluster nodes must be set at start, the launched cluster architectures are currently static and are not changeable during runtime.

### Comparison with similar software packages

To date, several approaches exist to improve the usability of currently available bioinformatic solutions. Systems such as Galaxy [14,15], GenePattern [16], Ergatis [17], Mobyle [18] and Taverna [19,20] try to facilitate the creation, execution and maintainability of workflows in a fast and user-friendly way. In contrast to these existing workflow platforms, Cloudgene’s primary focus lies on the usability of MapReduce jobs in public and private clouds for bioinformatic applications.

Galaxy CloudMan [21] is a similar approach to Cloudgene-Cluster and supports users to set up cloud clusters using Amazon EC2. It works in combination with Bio-Linux ( http://nebc.nerc.ac.uk/tools/bio-linux) and configures at start up the Oracle Grid Engine [22] as well as Galaxy. Unfortunately, CloudMan does not support MapReduce by default and therefore a graphical execution and monitoring of jobs is not possible.

Another mentionable and useful system is Amazon Elastic MapReduce (EMR) [9], which provides the opportunity to create job-flows including custom jars, streaming or Hive/Pig programs. Since everything is located on Amazon directly, a highly optimized version of Hadoop MapReduce in combination with Amazon S3 is provided and can be executed by a comprehensive user interface. Nevertheless, Amazon Elastic MapReduce can only be used in combination with Amazon EC2, sometimes preventing research institutes from using it due to data security rules or the enormous amount of data to transfer^3^ from their own institutional cloud. In contrast, Cloudgene allows launching MapReduce jobs both on public or private clouds, thereby enabling the user to define the location of data. Since Cloudgene does not utilize EMR for its job execution, additionally financial costs for EMR can be saved. Table 2 summarizes the comparison of Cloudgene and EMR and shows that Cloudgene is competitive regarding cluster set-up, job execution and data transfer.

CloVR [23] is a virtual image that provides several analysis pipelines to use on a personal computer as well as on the cloud. It utilizes the Grid Engine ( http://gridengine.org) for job scheduling and plans a possible future integration of Hadoop MapReduce. Eoulsan [24] is a modular framework which enables the setup of cloud computing clusters and automates the analysis of high throughput sequencing data. A modular plug-in system allows the integration of available algorithms. Eoulsan uses EMR for the execution of their MapReduce jobs and has to be executed on the command-line. Both systems improve the usage of programs for scientists, but are not focused on a graphical execution of jobs on public and private clusters.

### Future work

The success of a platform like Cloudgene goes hand in hand with the amount of involved users and scenarios. Therefore, our short-term focus will be on an extension of Cloudgene with new case scenarios, hopefully motivating users integrating their own MapReduce programs or systems. One of the biggest advantages of public clouds is the opportunity to rent as many computer nodes and thus computational power as needed. Thus, the next version of Cloudgene is conceived to provide functions for adding and removing computer nodes during runtime. Furthermore, a simple user interface for Hadoop is not only useful for end users but also for developers. It supports them during prototyping and testing of novel MapReduce programs by highlighting performance bottlenecks. Thus, we plan to implement time measurements on the map, reduce and shuffle phase and to visualize them in an intuitive chart. Additionally, Hadoop plans in its future version the support of alternate programming paradigms, which is particularly important for applications where custom frameworks outperform MapReduce by an order of magnitude (e.g. iterative applications like K-Means).

## Conclusions

We presented Cloudgene, a platform that allows scientists to set up a user-defined cluster in the cloud and to execute or monitor MapReduce jobs via a dynamically created web interface on the cluster. Cloudgene’s aim is to integrate existing and future MapReduce programs via a manifest file into one centralized platform. Cloudgene supports users without deeper background in computer science and improves the usability of currently available MapReduce programs in the field of Bioinformatics. We think that this approach improves the utilization of programs and the reproducibility of results. Additionally, we showed on different scenarios how an integration can be fulfilled without adding overhead to the computation, thereby improving both the development and the usability of a program.

## Availability and requirements

**Project name:** Cloudgene

**Project home page:**http://cloudgene.uibk.ac.at

**Operating System:** Cloudgene-Cluster (platform-independent), Cloudgene-MapRed (GNU/Linux)

**Programming language:** Java, JavaScript

**Other requirements:** Java 1.6, AWS-EC2 Account for public clouds, Hadoop MapReduce for private clouds

**License:** GNU GPL v3

**Any restrictions to use by non-academics:** None

## Endnotes

^1^ See http://cloudgene.uibk.ac.at/usecases.

^2^http://www.bioinformatics.babraham.ac.uk/projects/fastqc/.

^3^ Amazon Web Services (AWS) provides possibility to ship data on hard-drives.

^4^ In-house implementation.

## Competing interests

The authors declare that they have no competing interests.

## Authors' contributions

LF and SS initialized the project and developed Cloudgene. LF, SS, HW and AK-B were responsible for designing Cloudgene. AK-B, GS and FK supervised the project. LF, SS, HW and AK-B drafted the manuscript. All authors read and approved the final manuscript.

## Supplementary Material

Additional file 1 Supplementary Material to Cloudgene: A graphical execution platform for MapReduce programs on private and public clouds. Click here for file
