# Supplementary material for: Cloudgene: A graphical execution platform for MapReduce programs on private and public clouds
Source: BMC Bioinformatics. 2012 Aug 13;13:200. doi: 10.1186/1471-2105-13-200 (PMC3532373; doi:10.1186/1471-2105-13-200)
Supplement: Additional file 1 — Supplementary Material to Cloudgene: A graphical execution platform for MapReduce programs on private and public clouds. [file 1471-2105-13-200-S1.docx]

# Cloudgene: A graphical execution platform for MapReduce programs on private and public clouds

# Supplementary Material

# CloudBurst

CloudBurst [[1](#_ENREF_1)] is a parallel read-mapping algorithm to map NGS data to the human genome and other reference genomes. It is implemented as a MapReduce program using Hadoop and can be executed with the following command:

hadoop jar cloudburst/CloudBurst.jar \

reference_genome reads results 36 36 3 0 1 240 \

48 24 24 128 16

In order to integrate CloudBurst into Cloudgene a configuration file has to be created to start a Hadoop cluster on Amazon EC2 using a standard Ubuntu Linux (ami-da0cf8b3) and with open Hadoop ports 80, 50030 and 50070. The corresponding YAML has the following structure:

cluster:

image: us-east-1/ami-da0cf8b3

type: m1.large,m1.xlarge

ports: 80,50030,50070

service: hadoop

installMapred: true

As CloudBurst has no graphical user interface, Cloudgene-MapRed is installed on the Amazon EC2 cluster as well and used it for user interactions. For this purpose the command above with its arguments must be specified as followed:

mapred:

jar: CloudBurst.jar

params: $reference $reads $output $min_read_len $max_read_len $k

$allowdifferences $filteralignment 240 48 24 24 128 16

inputs:

- id: reference

description: Reference Genome

type: hdfs-file

makeAbsolute: false

- id: reads

description: Reads

type: hdfs-file

makeAbsolute: false

- id: min_read_len

description: min length of reads

type: number

value: 36

- id: max_read_len

description: max length of reads

type: number

value: 36

- id: k

description: mismatches

type: number

value: 3

- id: allowdifferences

description: Allow Differences

type: list

values:

0: mismatches only

1: indels as well

value: 0

- id: filteralignment

description: Filter Alignments

type: list

values:

0: all alignments

1: only report unambiguous best alignment

value: 1

outputs:

- id: output

description: Output Folder

type: hdfs-folder

download: true

mergeOutput: false

# Crossbow

Crossbow [[2](#_ENREF_2)] is a scalable software pipeline for whole genome resequencing analysis. It combines Bowtie, an ultrafast and memory efficient short read aligner, and SoapSNP, and an accurate genotyper. These tools are combined in an automatic, parallel pipeline.

In order to integrate Crossbow into Cloudgene, a configuration file to start a Hadoop cluster on Amazon EC2 has to be created using the CloudBioLinux image (ami-31bc7758) with open Hadoop ports 80, 50030 and 50070. The install.sh init-script downloads and installs all required software (e.g sratoolkit). The corresponding YAML has the following structure:

name: Crossbow

category: Genetics

version: 1.1.2

website: http://bowtie-bio.sourceforge.net/crossbow

author: Ben Langmead et al.

cluster:

image: us-east-1/ami-31bc7758

type: m1.large,m1.xlarge

ports: 80,50030,50070

user: ubuntu

service: hadoop

installMapred: true

initScript: install.sh

For Crossbow a web interface has already been made available by the authors. Nevertheless, by integrating these programs into Cloudgene, the users still benefit from (1) a standardized way to import/export data, (2) a system which keeps track of all previous executed workflows including the complete configuration set-up (input/output parameters, execution times, results) and (3) the possibility to concatenate different MapReduce jobs to pipelines. In this example, Cloudgene's concatenation functionality (specified as "steps" in the manifest file) has been used to execute several computation steps of Crossbow. This can be done by defining the output directory of step x (e.g. step 1: Pre-processing) as the new input directory for step x+1 (e.g. step 2: Alignment) in the manifest file. Even if the newly created workflow consists of several steps in the manifest file, the user can start it as one job.

mapred:

steps:

- name: Preprocess

mapper: Copy.pl --compress=gzip --stop=0 --maxperfile=500000 --s

--push=$temp/preproc

params: -input $manifest

-output $temp/preproc

-inputformat org.apache.hadoop.mapred.lib.NLineInputFormat

-numReduceTasks 0

-file Copy.pl

-file Get.pm

-file Counters.pm

-file Util.pm

-file Tools.pm

-file AWS.pm

- name: Alignment

mapper: Align.pl --discard-reads=0 --ref=$reference --destdir=/tmp/$job_id

--partlen=1000000 --qual=phred33 --truncate=0 -- --partition 1000000

--mm -t --hadoopout --startverbose -M 1

params: -input $temp/preproc

-output $temp/align

-numReduceTasks 0

-file Align.pl

-file Get.pm

-file Counters.pm

-file Util.pm

-file Tools.pm

-file AWS.pm

- name: Call SNPs

mapper: /bin/cat

reducer: Soapsnp.pl --discard-ref-bins=0 --refjar=$reference

--destdir=/tmp/$job_id --args=-2_-u_-n_-q --haploid_args=-r_0.0001

--diploid_args=-r_0.00005_-e_0.0001 --basequal=! --partition=1000000

--haploids=all --replace-uscores

params: -D stream.num.map.output.key.fields=3

-D mapred.text.key.partitioner.options=-k1,2

-input $temp/align

-output $temp/snp

-numReduceTasks 32

-partitioner org.apache.hadoop.mapred.lib.KeyFieldBasedPartitioner

-file Soapsnp.pl

-file Get.pm

-file Counters.pm

-file Util.pm

-file Tools.pm

-file AWS.pm

- name: Postprocess

mapper: /bin/cat

reducer: CBFinish.pl --cmapjar=$reference --destdir=/tmp/$job_id

--output=$output

params: -D stream.num.map.output.key.fields=2

-D mapred.text.key.partitioner.options=-k1,1

-input $temp/snp

-output $tempignoreme2

-numReduceTasks 30

-partitioner org.apache.hadoop.mapred.lib.KeyFieldBasedPartitioner

-file CBFinish.pl

-file Get.pm

-file Counters.pm

-file Util.pm

-file Tools.pm

-file AWS.pm

inputs:

- id: manifest

description: Manifest File

type: hdfs-file

- id: reference

description: Reference

type: hdfs-file

outputs:

- id: output

description: Output Folder

type: hdfs-folder

mergeOutput: false

download: true

zip: false

- id: temp

description: Temp

type: hdfs-folder

download: false

temp: true

After the tools folder has been uploaded to the cluster, the user starts a web browser by using the provided URL to (1) login to Cloudgene, (2) start up a cluster preconfigured with Crossbow and (3) run and monitor jobs.

**Myrna**

As Myrna [[3](#_ENREF_3)] implements several analysis steps, it is integrated in the same way as Crossbow. The Cloudgene-manifest-file has the following structure:

name: MyRNA

version: 1.1.2

category: Genetics

website: http://bowtie-bio.sourceforge.net/myrna

author: Ben Langmead et al.

cluster:

image: us-east-1/ami-31bc7758

type: m1.large,m1.xlarge

ports: 80,50030,50070

user: ubuntu

service: hadoop

installMapred: true

initScript: install.sh

mapred:

steps:

- name: Preprocessing

mapper: Copy.pl --compress=gzip --stop=0 --maxperfile=500000

--s --push=$temp/preproc

params: -input $manifest

-output $temp/preproc

-inputformat org.apache.hadoop.mapred.lib.NLineInputFormat

-numReduceTasks 0

-file Copy.pl

-file Get.pm

-file Counters.pm

-file Util.pm

-file Tools.pm

-file AWS.pm

- name: Alignment

mapper: Align.pl --discard-reads=$discard_fraction --ref=$reference

--destdir=/tmp/$job_id --partlen=1000000 --qual=$quality

--truncate=$trunctate_length --globals=$temp/globals

--discard-mate=$discard_mate --pool-trim-length=0 --

--partition -1000000 --mm -t --hadoopout --startverbose $bowtie_args

params: -input $temp/preproc

-output $temp/align

-numReduceTasks 0

-file Align.pl

-file Get.pm

-file Counters.pm

-file Util.pm

-file Tools.pm

-file AWS.pm

- name: Olaps

mapper: /bin/cat

reducer: Assign.pl --ivaljar=$reference --maxalns=350000 --partbin=200

--influence=1 --ival-model=ui --globals=$temp/globals --binwidth=0

--destdir=/tmp/$job_id --globals=$temp/globals

params: -D stream.num.map.output.key.fields=3

-D mapred.text.key.partitioner.options=-k1,2

-input $temp/align

-output $temp/olaps

-partitioner org.apache.hadoop.mapred.lib.KeyFieldBasedPartitioner

-numReduceTasks 32

-file Assign.pl

-file Assign.R

-file Get.pm

-file Counters.pm

-file Util.pm

-file Tools.pm

-file AWS.pm

- name: Normal

mapper: /bin/cat

reducer: Normal.pl --normal=lup --output=$temp/count

params: -D stream.num.map.output.key.fields=2

-D mapred.text.key.partitioner.options=-k1,1

-input $temp/olaps

-output $temp/normal

-partitioner org.apache.hadoop.mapred.lib.KeyFieldBasedPartitioner

-numReduceTasks 16

-file Normal.pl

-file Get.pm

-file Counters.pm

-file Util.pm

-file Tools.pm

-file AWS.pm

- name: Stats

mapper: /bin/cat

reducer: Stats.pl --family=$family --errdir=/home/hadoop/

--globals=$temp/globals --destdir=/tmp/$job_id --add-fudge=0

--nulls=$nulls

params: -D stream.num.map.output.key.fields=2

-D mapred.text.key.partitioner.options=-k1,1

-input $temp/normal

-output $temp/stats

-partitioner org.apache.hadoop.mapred.lib.KeyFieldBasedPartitioner

-numReduceTasks 32

-file Stats.pl

-file Stats.R

-file Get.pm

-file Counters.pm

-file Util.pm

-file Tools.pm

-file AWS.pm

- name: Summarize

mapper: /bin/cat

reducer: Summarize.pl --top=$top --nulls=$nulls --chosen-genes=$temp/chosen

params: -D stream.num.map.output.key.fields=2

-D mapred.text.key.partitioner.options=-k1,1

-input $temp/stats

-output $temp/summ

-partitioner org.apache.hadoop.mapred.lib.KeyFieldBasedPartitioner

-numReduceTasks 1

-file Summarize.pl

-file Get.pm

-file Counters.pm

-file Util.pm

-file Tools.pm

-file AWS.pm

- name: Postprocess

mapper: PostprocessMap.pl --destdir=/tmp/$job_id

--chosen-genes=$temp/chosen

reducer: PostprocessReduce.pl --ivaljar=$reference --cores=8

--destdir=/tmp/$job_id --output=$output --counts=$temp/count --minus-log

params: -D stream.num.map.output.key.fields=3

-D mapred.text.key.partitioner.options=-k1,2

-input $temp/summ,$temp/normal

-output $temp/ignoreme2

-partitioner org.apache.hadoop.mapred.lib.KeyFieldBasedPartitioner

-numReduceTasks 1

-file PostprocessMap.pl

-file PostprocessReduce.pl

-file Postprocess.R

-file Get.pm

-file Counters.pm

-file Util.pm

-file Tools.pm

-file AWS.pm

inputs:

- id: manifest

description: Manifest File

type: hdfs-file

- id: reference

description: Reference

type: hdfs-file

- id: quality

description: Quality encoding

type: list

value: solexa64

values:

phred33: Phred+33

phred64: Phred+64

solexa64: Solexa+64

- id: trunctate_length

description: Truncate length

type: number

value: 0

- id: discard_fraction

description: Discard fraction

type: number

value: 0

- id: top

description: Genes to report in detail

type: number

value: 50

- id: bowtie_args

description: Bowtie options

type: text

value: -m 1

- id: family

description: Model family

type: list

value: poisson

values:

poisson: Poisson

gaussian: Gaussian

- id: nulls

description: Null permutations

type: number

value: 0

- id: gene_footprint

description: Gene Intervals

type: list

value: intersect

values:

union: Union of exons

intersect : Intersection of transcripts

- id: pool_tech_reps

description: Pool technical replicates

type: checkbox

values:

true: --pool-reps

false:

- id: pool_reps

description: Pool all replicates

type: checkbox

values:

true: --pool-tech-reps

false:

- id: discard_mate

description: For paired-end reads, use just one mate

type: checkbox

value: 0

values:

true: 2

false: 0

outputs:

- id: output

description: Output Folder

type: hdfs-folder

mergeOutput: false

download: true

- id: temp

temp: true

type: hdfs-folder

download: false

# HaploGrep

HaploGrep [[4](#_ENREF_4)] is a reliable algorithm implemented in a web application to determine the haplogroup affiliation of thousands of mitochondrial DNA (mtDNA) profiles genotyped for the entire mtDNA or any part of it. As HaploGrep provides its own web interface we do not need to install Cloudgene-MapRed. Since it does not use the Hadoop service either, we noted this option in the configuration as well. HaploGrep listens on port 80 (http) and 443 (https), therefore these ports are marked in the YAML configuration. The configuration file for Cloudgene with all requirements looks as follows:

name: Haplogrep

description: Haplogrep

category: Genetics

cluster:

image: us-east-1/ami-da0cf8b3

type: m1.large,m1.xlarge

ports: 80

creationOnly: false

service: hadoop

installMapred: false

After the cluster setup is finalized, Cloudgene returns a web address which points to the installed instance of HaploGrep.

# CloudBioLinux

CloudBioLinux [[5](#_ENREF_5)] is an image for Amazon EC2 or Eucalyptus with preinstalled biological software, programming libraries and data sets. The freely available image can be started on an Amazon EC2 instance and provides the possibility to work on it via a graphical remote desktop. The configuration file for Cloudgene looks as follows:

name: CloudBioLinux

description: Using CloudBioLinux (Ubuntu 10.04)

category: Genetics

cluster:

image: us-east-1/ami-31bc7758

type: t1.micro,m1.large,m1.xlarge

ports: 80,50030,50070

user: ubuntu

service: hadoop

installMapred: true

creationOnly: false

# References

1. Schatz MC: **CloudBurst: highly sensitive read mapping with MapReduce**. *Bioinformatics* 2009, **25**(11):1363-1369.

2. Langmead B, Schatz MC, Lin J, Pop M, Salzberg SL: **Searching for SNPs with cloud computing**. *Genome Biol* 2009, **10**(11):R134.

3. Langmead B, Hansen KD, Leek JT: **Cloud-scale RNA-sequencing differential expression analysis with Myrna**. *Genome biology* 2010, **11**(8):R83.

4. Kloss-Brandstätter A, Pacher D, Schönherr S, Weissensteiner H, Binna R, Specht G, Kronenberg F: **HaploGrep: a fast and reliable algorithm for automatic classification of mitochondrial DNA haplogroups**. *Hum Mutat* 2011, **32**(1):25-32.

5. Krampis K, Booth T, Chapman B, Tiwari B, Bicak M, Field D, Nelson K: **Cloud BioLinux: pre-configured and on-demand bioinformatics computing for the genomics community**. *BMC Bioinformatics* 2012, **13**(1):42.
